# Combination of pembrolizumab and imatinib in a patient with double KIT mutant melanoma: A case report

**DOI:** 10.1097/MD.0000000000017769

**Published:** 2019-11-01

**Authors:** Yara Abdou, Ankita Kapoor, Lamya Hamad, Marc S. Ernstoff

**Affiliations:** a Roswell Park Comprehensive Cancer Center, Buffalo; b Rochester General Hospital, Rochester, NY.

**Keywords:** imatinib, immunotherapy, KIT mutation, liver toxicity, melanoma, PD1-inhibition, pembrolizumab

## Abstract

**Rationale:**

The treatment of metastatic melanoma has been revolutionized in the past decade because of the development of immunotherapies and targeted therapies. Despite these developments, there is still an unmet clinical need for more advanced combination therapies for the subset of patients who remain resistant to immunotherapy or targeted therapy alone. To our knowledge, no reports have been published on combinations of PD-1 blockades and c-KIT inhibitors in melanoma patients. Furthermore, data are limited regarding the safety and efficacy of this combination in patients harboring KIT mutations.

**Patient concerns and diagnosis:**

We report a case of an 82-year-old female with metastatic melanoma who was found to have double KIT mutations at V559 and N822I.

**Interventions:**

She was treated with a combination of c-KIT inhibitor and PD-1 blockade after being resistant to anti-PD-1 monotherapy.

**Outcomes:**

Patient developed two episodes of grade 2 liver toxicity requiring treatment breaks followed by a dose reduction. Her transaminitis eventually resolved and patient remained on combination treatment for almost two years with good control of her disease prior to progression.

**Lessons:**

Treatment options for patients who progress after PD-1 inhibitors are very limited; therefore, there is a high unmet clinical need for this patient population. Combining Imatinib with checkpoint inhibitors may be efficacious in patients with metastatic melanoma and KIT mutations. This novel combination can cause additional toxicities which seem to be overall manageable.

## 1 Introduction

Melanoma accounts for only about 1% of all skin cancers, yet it is responsible for a large majority of skin cancer deaths because of its aggressive nature. The treatment of metastatic melanoma has been revolutionized in the past decade because of the development of agents that target elements in the immune system including cytotoxic T-lymphocyte-associated protein 4 (CTLA-4), programmed cell death protein-1 (PD-1) and programmed cell death protein ligand 1 (PD-L1).^[1]^ Checkpoint inhibitors (CPI) have played a fundamental role in melanoma treatment; however, many patients remain refractory. On the other hand, identification of targetable mutations in the mitogen-activated protein kinases (MAPK) pathway have also led to new therapies in melanoma. The most frequently activating mutation is in the BRAF kinase representing about 35% to 40% of melanomas.^[2]^ The receptor tyrosine kinase c-Kit is less frequently mutated and seen mostly in mucosal and acral melanomas, and less often in melanomas with chronic sun-damaged skin.^[3]^ The activating mutations can be blocked effectively with targeted therapies such as Imatinib.^[4]^

Despite these developments, there is still an unmet clinical need for more advanced combination therapies for the subset of patients who remain resistant to check point inhibitors or targeted therapy alone. Therefore, it is essential to determine ways of combining these therapies and exploring potential toxicities that may arise. The ultimate goal is to discover potential novel combinations with a tolerable safety profile and enhanced efficacy.

Here, we report a case of an 82-year-old woman with metastatic cutaneous melanoma, harboring double KIT mutations, that was treated with Imatinib and anti-PD-1 therapy after being resistant to anti-PD-1 monotherapy. This is the first time to our knowledge that this novel combination has been reported in metastatic melanoma.

## 2 Case report

An 82-year-old woman with past medical history of hypertension and hyperlipidemia, presented with a right cheek nodule in December 2015. Biopsy result was consistent with ulcerated malignant melanoma, nodular type, Breslow depth of at least 2.7 mm. The nodule was locally resected, pathology results were consistent with extensive residual malignant melanoma, Breslow depth of 6.05 mm with multiple microsatellite metastases present, angiolymphatic invasion was identified and one sentinel lymph node was negative for melanoma (0/1). Melanoma in situ extended to the superior margin and medial and lateral specimen tips, therefore procedure was followed with Mohs surgery which showed no residual disease. Six months later, patient presented with two new nodules on her right cheek below the area of her prior excision. At that time, she was also noted to have right submandibular lymphadenopathy. Excisional biopsy of the nodule and FNA of submandibular lymph node showed recurrent malignant melanoma. Staging CT chest/abdomen/pelvis and brain MRI showed no evidence of distant metastatic disease. Multidisciplinary teams agreed on deferring surgery and proceeding with systemic therapy. Patient was subsequently started on anti-PD-1 therapy with Pembrolizumab at a fixed dose of 200 mg every 3 weeks. Patient continued to show a positive response until fourteen months of therapy when restaging CT scans demonstrated a left upper lobe nodule concerning for metastatic disease. PET scan demonstrated a high SUV uptake of 15, also consistent with metastatic disease. A left lung needle biopsy was subsequently completed which was consistent with metastatic malignant melanoma. Mutational analysis revealed double KIT mutations at V559 and N822I, therefore, Imatinib; a tyrosine kinase inhibitor known as Gleevec, was started at a dose of 400 mg daily. This was given in combination with Pembrolizumab at 200 mg every three weeks. Shortly after, patient developed a pruritic rash on her back and shoulders in addition to Grade 2 transaminitis. Imatinib was held for approximately 3 weeks until her symptoms and transaminitis resolved. No further intervention was needed. She then restarted Imatinib at half dose of 200 mg daily. One month later, patient developed another episode of grade 2 transaminitis. Imatinib was held for approximately three weeks and once her LFTs normalized, Imatinib was restarted at 400 mg 3 times a week (Monday/Wednesday/Friday). Restaging scans completed after 3 months of combination therapy showed significant decrease in size of lung nodule. In 6 months, restaging scans showed complete remission with no radiographical evidence of metastatic disease. Patient continued on combination therapy of Pembrolizumab and Imatinib, with no evidence of disease for almost 12 months. However, she ultimately developed recurrent disease with brain metastases after a total of 2 years on combination therapy. She was eventually transitioned to hospice care. Patient's health care proxy has provided informed consent for publication of the case

## 3 Discussion

Immunotherapy, particularly blockade of the inhibitory receptor, PD-1 or the ligand; PD-L1, has shown effectiveness in a variety of cancers, but in many patients this effect remains of transient importance due to development of resistance.

Emerging data has shown that the tumor microenvironment is important for the effectiveness of immunotherapy.^[5]^ Different approaches to modulate the tumor microenvironment have been pursued, including oncolytic modified herpes simplex virus, which has also been approved for the treatment of melanoma.^[6]^ The ultimate goal is to create an immunogenic environment by increasing tumor-infiltrating lymphocytes and tumor antigen recognition.

In our case, we report the first use of combination c-KIT inhibitor and PD-1 blockade in an attempt to overcome anti-PD1 resistance.

c-KIT is a type III receptor tyrosine kinase (RTK), which is involved in intracellular signaling and plays a significant role in cancer occurrence.^[7]^ Previous studies have shown that point mutations in *c-KIT* result in constitutive activation of the c-KIT protein in melanoma cells, and this lead to activation of downstream proliferative and prosurvival signaling pathways. In vitro studies also showed that treatment with Imatinib, a tyrosine kinase inhibitor, led to apoptosis of melanoma cells.^[8,9]^

In experiments conducted in mouse models by Seifert et al, it has been shown that there is increased proliferation of intratumoral CD8+ T cells while inducing apoptosis of regulatory T cells, when a combination therapy of Imatinib and PD-1/PD-L1 blockade was used.^[10]^ This in vivo model suggests that the combination therapy could have a role in altering the tumor microenvironment by changing the tumor from cold to hot, and ultimately making it more responsive to immunotherapy.

Combinations of targeted therapy and immunotherapy have been safely reported with dual MAPK inhibitors and anti-PD1, however increased liver toxicities were seen when MAPK inhibitors were given with anti-CTLA4 agents.^[11]^ To our knowledge, no reports have been published on combination of c-KIT inhibitor and PD-1 blockade.

Previous clinical trials with Imatinib have established that Imatinib is a relatively safe drug with fewer side effects profile.^[12]^ Side effects are generally mild to moderate; the most common being: fluid retention, diarrhea, nausea, fatigue, rash, and muscle cramps, which can be managed effectively by either dose modifications or supportive care medicines. There is also the risk of more severe symptoms, though not common, such as liver toxicity, hemorrhage, and upper respiratory tract infections.^[13]^ The patient described in our case study experienced grade 2 liver toxicity. For elevations of transaminases >5 IULN, Imatinib should be held until resolution to <2.5 IULN and restarted at a lower dose. The recommended dose reduction is 25% or 300 mg^[14]^; however, this patient was dose reduced by 50% mainly due to being on combination therapy with anti-PD1 which is also known to cause autoimmune hepatitis. It is difficult to determine which agent is the immediate cause of this patient's liver injury. However, it is reasonable to assume that the combination of both agents has made this event more likely.

There are no current guidelines for dealing with Imatinib side effects while on combination therapy with checkpoint inhibitors. To date, approximately 10 registered clinical trials have explored the safety and efficacy of Imatinib alone or with other agents in metastatic melanoma, however majority of the studies were not successfully completed.^[13]^ One of which was an early phase trial of Pembrolizumab and Imatinib in patients with c-Kit mutations. The trial was withdrawn due to low accrual.

We report the first patient treated with combination Imatinib and pembrolizumab demonstrating that Imatinib toxicity may be increased but with close monitoring and dose modification can be managed successfully.

## 4 Conclusion

Treatment options for patients who progress after PD-1 inhibitors are very limited; therefore, there is a high unmet clinical need for this patient population. The addition of Imatinib to anti-PD1 therapy may create a favorable tumor microenvironment which enhances antitumor activity and subsequently improving efficacy of checkpoint inhibitors. This novel combination may cause additional toxicities that are overall manageable. Further information is needed on how to deal with serious side effects while on combination therapy.

## Author contributions

**Resources:** Yara Abdou.

**Supervision:** Yara Abdou, Marc S. Ernstoff.

**Writing – original draft:** Yara Abdou, Ankita Kapoor.

**Writing – review & editing:** Yara Abdou, Ankita Kapoor, Lamya Hamad, Marc S. Ernstoff.

Yara Abdou orcid: 0000-0002-4827-8613.
